# Gender differences in GPs’ strategies for coping with the stress of the COVID-19 pandemic in Catalonia: A cross-sectional study

**DOI:** 10.1080/13814788.2022.2155135

**Published:** 2022-12-19

**Authors:** Enric Aragonès, Maribel Fernández-San-Martín, Maria Rodríguez-Barragán, Francisco Martín-Luján, Mònica Solanes, Anna Berenguera, Antoni Sisó, Josep Basora

**Affiliations:** a Primary Care Research Institute IDIAP Jordi Gol, Barcelona, Spain; b Catalan Society of Family and Community Medicine CAMFiC, Barcelona, Spain; c Primary Healthcare Service Camp de Tarragona, Catalan Health Institute, Tarragona, Spain; d Primary Care Barcelona city, Catalan Health Institute, Barcelona, Spain; e Primary Care Centre ‘La Mina’, Catalan Health Institute, Barcelona, Spain; f Faculty of Medicine and Health Sciences, University Rovira i Virgili, Reus, Spain; g 11 de Setembre’ Primary Care Centre, Catalan Health Institute, Lleida, Spain; h Consorci d’Atenció Primària de Salut Barcelona Esquerra (CAPSBE), Barcelona, Spain

**Keywords:** Coping strategies, stress, Covid-19 pandemic, general practitioner (GP), gender differences

## Abstract

**Background:**

The Covid-19 pandemic has increased stress levels in GPs, who have resorted to different coping strategies to deal with this crisis. Gender differences in coping styles may be contributing factors in the development of psychological distress.

**Objectives:**

To identify differences by gender and by stress level in coping strategies of GPs during the Covid-19 pandemic.

**Methods:**

A cross-sectional, web-based survey conducted with GPs in Catalonia (Spain), in June–July 2021. *via* the institution’s email distribution list, all GPs members of the Catalan Society of Family and Community Medicine were invited to complete a survey assessing sociodemographic, health and work-related characteristics, experienced stress (Stress scale of the Depression, Anxiety and Stress Scales-DASS 21) and the frequency of use of a range of coping strategies (Brief-COPE) classified as problem-focused, emotion-focused and avoidant strategies, some of which are adaptive and others maladaptive. We compared the scores of each strategy by gender and stress level using Student’s *t*-test.

**Results:**

Of 4739 members, 522 GPs participated in the study (response rate 11%; 79.1% women; mean age = 46.9 years, *SD* = 10.5). Of these, 41.9% reported moderate-severe stress levels. The most common coping strategies were acceptance, active coping, planning, positive reframing and venting. More frequently than men, women resorted to emotional and instrumental support, venting, distraction and self-blame, whereas men used acceptance and humour more commonly than women. Moderate-severe stress levels were associated with non-adaptive coping, with increased use of avoidance strategies, self-blame, religion and venting, and decreased use of positive reframing and acceptance.

**Conclusion:**

The most common coping strategies were adaptive and differed by gender. However, highly stressful situations caused maladaptive strategies to emerge.

KEY MESSAGES

- Coping strategies most commonly used by GPs to deal with the stress associated with the Covid-19 pandemic were adaptive.
- Coping strategies differ by gender: women seek more instrumental and emotional support than men and they also blame themselves more often than men.
- High-stress levels are associated with maladaptive coping strategies.

## Introduction

During the Covid-19 pandemic, health professionals and GPs specifically, have faced unprecedented uncertainty, overload, and changes of responsibilities and working conditions. Combined with insufficient resources and their own risk of infection, the prevalence of psychological distress [1], burnout, and mental disorders in primary care health workers spiked, particularly in women [2]. Gender differences include biological, social and demographic factors, as well as coping mechanisms [3].

Coping strategies are mental processes and behaviours used to manage stressful situations. Problem-focused coping involves behaviour geared towards modifying or eliminating sources of stress. Emotion-focused strategies aim to lessen the emotional consequences of stressful events. Avoidant strategies imply avoiding instead of managing stressors. Problem-focused coping strategies are generally more adaptive than emotion-focused and avoidant strategies [4].

Our objective is to identify and characterise the coping strategies used by GPs during the pandemic and to describe differences in coping patterns based on gender and stress levels.

## Methods

### Design

Cross-sectional evaluation of self-reported online surveys administered to GPs between 18 June and 28 July 2021.

### Population and sample

Doctors were invited to participate by email blasting members of the Catalan Society of Family Medicine (census sampling; *n* = 4739). Five reminder invitations were sent during the recruitment period.

### Measurements and variables

#### Coping strategies

We used the Brief-COPE [5] questionnaire to evaluate strategies for stress. It consists of 28 items that assess how often 14 different coping strategies are used. Responses are measured on a Likert scale ranging from ‘I haven’t been doing this at all’ (1 point) to ‘I’ve been doing this a lot’ (4 points).

The coping strategies are divided in:

- *Problem-focused strategies*: active coping, use of instrumental support, positive reframing, and planning;
- *Emotion-focused strategies*: use of emotional support, venting, humour, acceptance, religiosity, and self-blame; and
- *Avoidant strategies*: denial, self-distraction, substance use, and behavioural disengagement.

To classify the various strategies as adaptive or maladaptive we used the criteria of Eisenberg et al. [6]. According to their two-factor solution, adaptive coping consists of the subscales of acceptance, active coping, emotional support, instrumental support, planning, and positive reframing. Maladaptive coping comprises the subscales of behavioural disengagement, denial, self-blame, self-distraction, substance use, and venting. Humour and religion were excluded.

##### Experienced stress

Measured using the Depression, Anxiety and Stress Scales (DASS-21) [7], which consists of seven items evaluated according to frequency, from ‘it never happens to me’ (0 points) to ‘it always happens to me’ (3 points). The results are stratified into ‘no stress’ (0–7 points), ‘mild’ stress (8–9 points), ‘moderate stress’ (10–12 points), ‘severe stress’ (13–16 points) and ‘very severe stress’ (≥ 17). We have further categorised these results into two groups: ‘absent or mild’ stress (0–9 points), and ‘moderate to very severe’ stress (≥ 10 points).

##### Personal factors

To characterise the sample, we also measured age, gender, workplace (primary care vs. others), exposure to Covid-19 patients (no, occasional, frequent, very frequent), Covid-19 infection, and history of a mental disorder.

### Statistical analysis

We used Student’s *T* test to compare the mean scores of each coping strategy by gender in the overall sample and also to compare the strata representing different stress levels.

### Ethical aspects

Informed consent was obtained from participants. The study protocol was approved by the Research Ethics Committee of the IDIAP Jordi Gol (21/098-PCV).

## Results

### Sample characteristics

Of the 4739 members, 522 GPs (11%) provided valid responses to the survey. The target population was 73.3% female and the mean age was 44.4 years (*SD*: 12.0). Of all participants, 413 (79.1%) were women and one reported non-binary gender. The mean age was 46.9 years (*SD*: 10.5), 90.8% worked in primary care, 78.6% were frequently or very frequently in contact with Covid-19 patients, 24.7% had been infected with SARS-CoV-2, and 23 2% reported a history of mental health disorder. The only participant who self-identified as non-binary gender was 30 years old, worked in primary care, reported very frequent contact with Covid-19 patients at work, had been previously infected by Covid-19, and had a history of the disorder mental.

### Coping strategies

Coping strategies most often used by GPs (i.e. with a score > 2.5 in a range of 1–4) included problem-focused coping: active coping, positive reframing, and planning; emotion-focused coping: acceptance, seeking emotional support and venting; and an avoidance style: self-distraction. Other avoidance strategies were uncommon (i.e. scoring < 1.5): denial, behavioural disengagement, or substance use (Table 1).

**Table 1.** Coping strategies in the total sample and by gender.

	Total (*n* = 522)	Women (*n* = 413)	Men (*n* = 108)	
Coping strategies^a^	mean (*SD*)	mean (*SD*)	mean (*SD*)	*p*-Value^b^
Problem-focused coping				
Active coping	2.87 (0.65)	2.87 (0.64)	2.87 (0.67)	0.989
Use of instrumental support	2.42 (0.76)	2.47 (0.76)	2.21 (0.75)	**0.003**
Positive reframing	2.58 (0.76)	2.58 (0.76)	2.58 (0.79)	0.991
Planning	2.78 (0.65)	2.78 (0.65)	2.81 (0.67)	0.634
Emotion-focused coping				
Use of emotional support	2.57 (0.77)	2.61 (0.78)	2.40 (0.70)	**0.017**
*Venting*	2.60 (0.75)	2.68 (0.73)	2.28 (0.75)	**<0.001**
Humour	2.36 (0.92)	2.31 (0.89)	2.56 (1,01)	**0.013**
Acceptance	3.24 (0.62)	3.20 (0.61)	3.39 (0.65)	**0.006**
*Self-blame*	2.29 (0.67)	2.35 (0.68)	2.05 (0.61)	**<0.001**
Religion	1.65 (0.84)	1.68 (0.87)	1.51 (0.74)	0.060
Avoidance coping				
*Self-distraction*	2.52 (0.70)	2.58 (0.68)	2.28 (0.72)	**<0.001**
*Denial*	1.23 (0.49)	1.25 (0.50)	1.17 (0.45)	0.126
*Substance use*	1.21 (0.50)	1.20 (0.48)	1.27 (0.57)	0.208
*Behavioural disengagement*	1.35 (0.57)	1.37 (0.58)	1.29 (0.55)	0.190

^a^Coping strategies measured with the Brief-COPE questionnaire, ranging from 1 (I haven’t been doing this at all) to 4 (I’ve been doing this a lot); maladaptive strategies are in italics.

^b^Student’s *t*-test comparing women vs. men, *p* values <0.05 are marked in bold.

### Coping strategies by gender

We noted significant differences by gender (Table 1): Women sought more instrumental and emotional support, venting and self-distraction, and scored significantly higher on self-blame than men. Men presented greater acceptance and use of humour than women.

### Coping strategies by level of stress

Comparing the profiles of coping strategies by stress level (Table 2), results show that high stress was associated with higher scores on maladaptive strategies such as venting, self-blame, self-distraction, denial, behavioural disengagement, and substance use. In contrast, scores on adaptive strategies such as acceptance and problem reframing decreased.

**Table 2.** Coping strategies according to level of stress.

	No stress or mild stress^a^ (*n* = 299)	Moderate, severe or extremely severe stress^a^ (*n* = 216)	
Coping strategies^b^	mean (*SD*)	mean (*SD*)	*p*-Value^c^
Problem-focused coping			
Active coping	2.84 (0.63)	2.90 (0.67)	0.319
Use of instrumental support	2.37 (0.74)	2.48 (0.79)	0.111
Positive reframing	2.66 (0.75)	2.48 (0.77)	**0.010**
Planning	2.77 (0.65)	2.80 (0.66)	0.526
Emotion-focused coping			
Use of emotional support	2.55 (0.73)	2.60 (0.82)	0.524
*Venting*	2.53 (0.72)	2.70 (0.79)	**0.010**
Humour	2.38 (0.90)	2.34 (0.94)	0.653
Acceptance	3.33 (0.61)	3.11 (0.61)	**<0.001**
*Self-blame*	2.11 (0.57)	2.55 (0.73)	**<0.001**
Religion	1.57 (0.80)	1.74 (0.89)	**0.026**
Avoidance coping			
*Self-distraction*	2.40 (0.67)	2.68 (0.72)	**<0.001**
*Denial*	1.13 (0.34)	1.37 (0.62)	**<0.001**
*Substance use*	1.11 (0.34)	1.35 (0.63)	**<0.001**
*Behavioural disengagement*	1.19 (0.37)	1.57 (0.71)	**<0.001**

^a^Level of stress measured with the stress scale of the DASS-21 (Depression, Anxiety and Stress Scales).

^b^Coping strategies measured with the Brief-COPE questionnaire, ranging from 1 (I haven’t been doing this at all) to 4 (I’ve been doing this a lot); maladaptive coping strategies are in italics.

^c^Student’s *t*-test comparing the categories according to stress level, *p* values <0.05 are marked in bold.

### Coping strategies by gender according to stress level

Table 3 shows gender differences stratified by stress level. Differences in self-blame and distraction, more common in women, were aggravated in the moderate-severe stress layer, and the use of substances emerged as strategy in men.

**Table 3.** Coping strategies by gender according to level of stress.

	No stress or mild stress^a^			Moderate, severe or extremely severe stress^a^		
	Women (*n* = 225)	Men (*n* = 74)		Women (*n* = 185)	Men (*n* = 31)	
Coping strategies^b^	mean (*SD*)	mean (*SD*)	*p*-Value^c^	mean (*SD*)	mean (*SD*)	*p*-Value^c^
Problem-focused coping						
Active coping	2.85 (0.61)	2.84 (0.69)	0.969	2.90 (0.68)	2.94 (0.63)	0.774
Use of instrumental support	2.43 (0.71)	2.20 (0.79)	**0.022**	2.51 (0.81)	2.25 (0.66)	0.082
Positive reframing	2.65 (0.74)	2.69 (0.79)	0.698	2.50 (0.77)	2.34 (0.77)	0.282
Planning	2.75 (0.63)	2.83 (0.70)	0.352	2.81 (0.68)	2.77 (0.58)	0.723
Emotion-focused coping						
Use of emotional support	2.58 (0.74)	2.46 (0.69)	0.214	2.65 (0.83)	2.29 (0.70)	**0.026**
*Venting*	2.64 (0.69)	2.19 (0.69)	**0.000**	2.73 (0.78)	2.50 (0.85)	0.124
Humour	2.30 (0.86)	2.62 (0.98)	**0.007**	2.32 (0.92)	2.41 (1.07)	0.636
Acceptance	3.28 (0.60)	3.51 (0.58)	**0.004**	3.11 (0.60)	3.11 (0.72)	0.985
*Self-blame*	2.15 (0.56)	1.97 (0.56)	**0.018**	2.60 (0.72)	2.24 (0.68)	**0.011**
Religion	1.60 (0.82)	1.49 (0.73)	0.275	1.78 (0.91)	1.56 (0.77)	0.207
Avoidance coping						
*Self-distraction*	2.45 (0.65)	2.24 (0.68)	**0.016**	2.73 (0.69)	2.36 (0.80)	**0.007**
*Denial*	1.15 (0.37)	1,07 (0.21)	0.065	1.36 (0.61)	1.39 (0.70)	0.827
*Substance use*	1.11 (0.34)	1.12 (0.35)	0.763	1.31 (0.59)	1.59 (0.80)	**0.017**
*Behavioural disengagement*	1.19 (0.34)	1.19 (0.45)	0.978	1.58 (0.72)	1.50 (0.70)	0.563

^a^Level of stress measured with the stress scale of the DASS-21 (Depression, Anxiety and Stress Scales).

^b^Coping strategies measured with the Brief-COPE questionnaire, ranging from 1 (I haven’t been doing this at all) to 4 (I’ve been doing this a lot), maladaptive coping strategies are in italics.

^c^Student’s *t*-test comparing women vs. men, *p* values <0.05 are marked in bold.

## Discussion

### Main findings

The most commonly used coping strategies by GPs were adaptive, problem-focused (active coping, positive reframing, planning), or emotion-focused (acceptance, seeking emotional support). However, highly stressful situations increased the use of ineffective strategies (avoidance strategies, self-blame), and decreased adaptive strategies (acceptance of the problem, positive reframing). As anticipated, we found gender differences in stress-coping strategies.

### Comparison with existing literature

Women are more likely to use emotion-focused strategies, as described in other population groups [8]. Women doctors resorted to emotional support, particularly when suffering from high stress levels but also to seeking instrumental support, a problem-focused strategy. Emotional support refers to looking for empathy and understanding, and instrumental support to seeking specific advice and practical help. The strategy of seeking emotional and instrumental support is considered highly effective for managing stress in healthcare professionals [9]. However, high levels of stress exacerbated the difference between genders regarding self-blame. Self-blame has been associated with physicians’ burnout and psychological distress, particularly in women [10,11].

Substance abuse as an escape strategy increases in stressful situations, particularly in men. Even though this behaviour hinders effective coping, increase in substance abuse in health care workers during the pandemic has already been reported [12,13].

### Limitations

This study has limitations. Firstly, the possibility of self-selection bias of participants who completed the survey, although this limitation is inherent in this methodology [14]. Secondly, the cross-sectional design does not allow to infer causality from the factors associated with the outcomes. We are currently conducting a prospective follow-up of this cohort to assess the evolution of these variables in relation to the progress of the pandemic. Finally, while women seem overrepresented in this sample, it only reflects that most GPs are currently women [15].

### Implications

The need to recognise the distress of healthcare professionals during the pandemic should be linked to access to preventive and therapeutic resources and to a healthier organisation of work [16]. Our results can help design interventions to promote mental health in GPs, taking gender differences into account. We underscore the need to use effective strategies to cope with stress, such as providing mutual support within primary care teams and to preventing deleterious behaviours such as self-blame and substance use.

## Conclusion

During the Covid-19 pandemic, GPs primarily addressed stress with adaptive strategies, with patterns of use of coping strategies differing by gender. High-stress levels were associated with maladaptive coping strategies.
